# Supplementary material for: Cationic Fluorescent Nanogel Thermometers based on Thermoresponsive Poly(N-isopropylacrylamide) and Environment-Sensitive Benzofurazan
Source: Polymers (Basel). 2019 Aug 4;11(8):1305. doi: 10.3390/polym11081305 (PMC6723757; doi:10.3390/polym11081305)
Supplement: Supplementary file 1 [file polymers-11-01305-s001.pdf]

Supplementary Materials for

Cationic fluorescent nanogel thermometers based on thermoresponsive poly(*N*-isopropylacrylamide) and environment-sensitive benzofurazan

Teruyuki Hayashi, Kyoko Kawamoto, Noriko Inada, and Seiichi Uchiyama

Materials and Methods

Bulletized procedures corresponding to Subsections 2.2 and 2.4 are described below.

*2.2. Preparation of cationic nanogels (NANOGEL-**1** and NANOGEL-**2**) and cationic nanogel thermometers (NANOGEL-**3**~**6**).*

(i) Dissolve NIPAM (2 mmol), DBD-AA (0–20 μmol), MBAM (20 μmol), TMEDA (58 μmol), and/or CTAC (0 or 38 μmol) in 19 ml of water in a three-neck round-bottom flask.
(ii) Bubble dry Ar gas through the solution at 70 °C for 30 min.
(iii) Add ADIP (560 μmol) in 1 ml of water to the solution.
(iv) Stir the mixture using a rod with a paddle at 250 rpm and 70 °C for 1 h under an Ar atmosphere.
(v) Pour the mixture into 400 ml of water and add NaCl up to saturating.
(vi) Collect precipitated nanogels by filtration and dissolve them in approximately 10 ml of water.
(vii) Pour the solution into a dialysis membrane and dialyze it for at least one week.
(viii) Freeze-dry the dialyzed solution to obtain purified nanogels.

2.4. Introduction of cationic fluorescent nanogel thermometers into HeLa cells.

(A) Standard procedure (to introduce nanogel thermometers into adherent HeLa cells)
 (i) Prepare the HeLa cell culture in a 35-mm glass-bottom dish at 30–50% confluence.
 (ii) Remove the DMEM from the cell culture dish with a pipette.
 (iii) Rinse the HeLa cells with 1 ml of 1×PBS.
 (iv) Replace the 1×PBS by 1 ml of 0.05 w/v% nanogel thermometers in a 5 w/v% glucose solution.
 (v) Incubate the dish at 25 or 37 °C for 5, 10, or 20 min.
 (vi) Remove the nanogel thermometers solution from the dish and rinse the cells with 1 ml of 1×PBS three times.
 (vii) Add 2ml of phenol red-free DMEM to the dish.
(B) Modified procedure (to introduce nanogel thermometers into suspended HeLa cells)
 (i) Prepare the HeLa cell culture in a 100-mm glass-bottom dish at 70–100% confluence.
 (ii) Remove the DMEM from the cell culture dish with a pipette.
 (iii) Rinse the HeLa cells with 1 ml of 1×PBS and remove the 1×PBS.
 (iv) Add 0.5 ml of a 0.05 w/v% trypsin-EDTA-1×PBS solution to the dish and incubate the dish at 37 °C for 3–5 min.
 (v) Add 9 ml of DMEM to the dish and suspend the detached HeLa cells.

(vi) Transfer 1 ml of the cell suspension to 1.5 ml tube.
(vii) Centrifuge the cell suspension at 1,200 rpm at 4 °C for 1 min and discard the supernatant.
(viii) Rinse cells with 1 ml of 1×PBS.
(ix) Repeat steps (vii) and (viii) twice, then step (vii).
(x) Suspend the cells in 1 ml of 0.05 w/v% nanogel thermometers in a 5 w/v% glucose solution.
(xi) Incubate the cell suspension at 25 °C for 20 min without CO_2 supply.
(xii) Centrifuge the cell suspension at 1,200 rpm at 4 °C for 1 min and discard the supernatant (probe solution).
(xiii) Rinse the cells with 1 ml of 1×PBS.
(xiv) Repeat steps (xii) and (xiii) twice, and step (xii).
(xv) Suspend the collected HeLa cells in 10 ml of DMEM.
(xvi) Add 2 ml of the cell suspension to a 35-mm glass bottom dish and incubate it at 37 °C with 5% CO_2 for one or two nights before observation.

NANOGEL-**1**

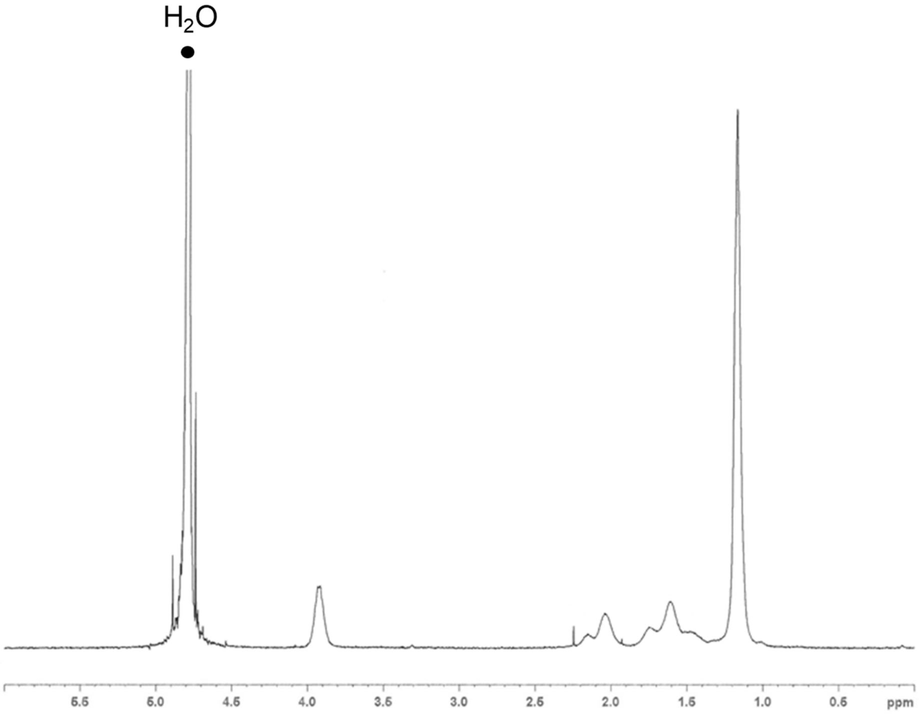

NANOGEL-**2**

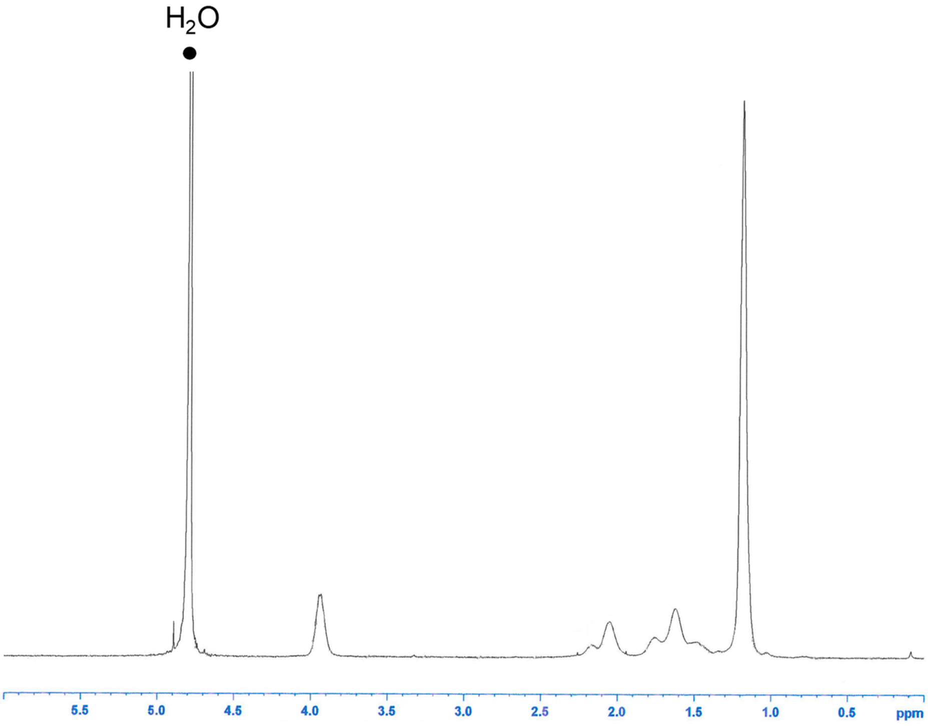

Figure S1. ^{1}H-NMR charts of NANOGEL-**1**~**6**.

NANOGEL-**3**

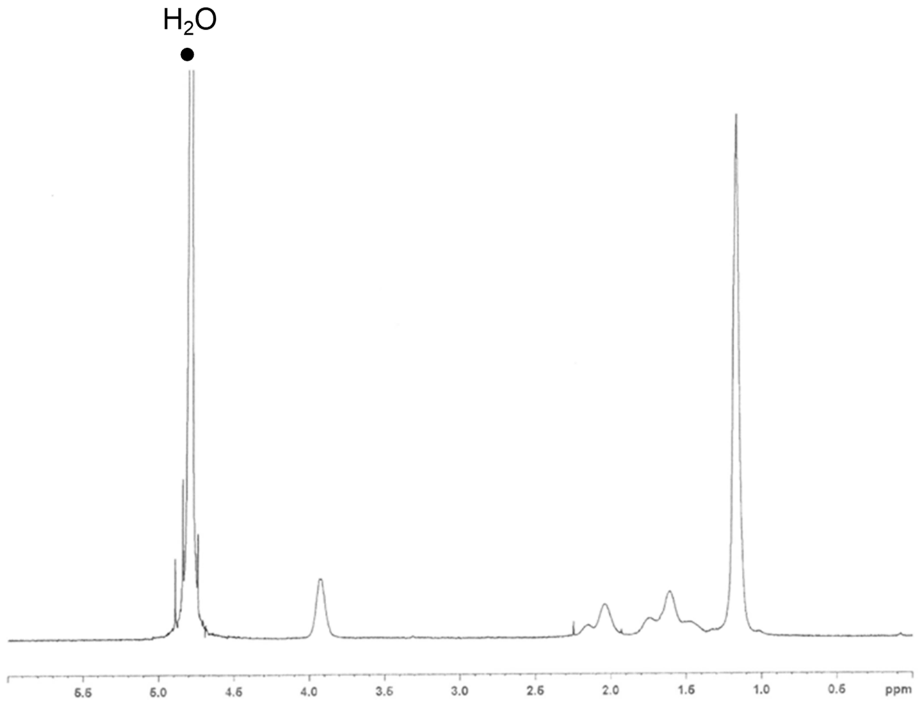

NANOGEL-**4**

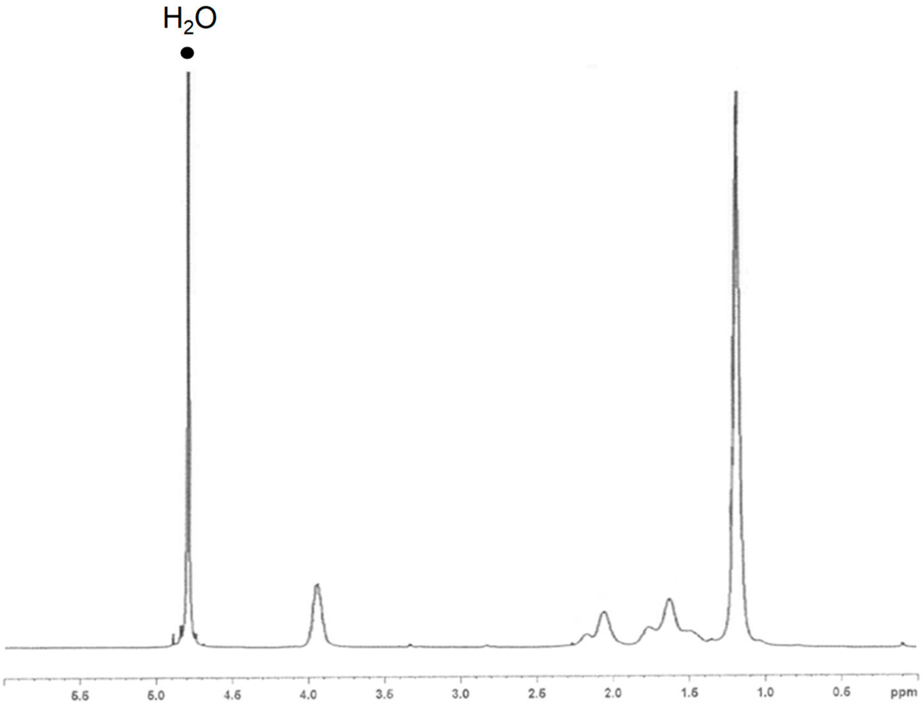

Figure S1. (continued)

NANOGEL-**5**

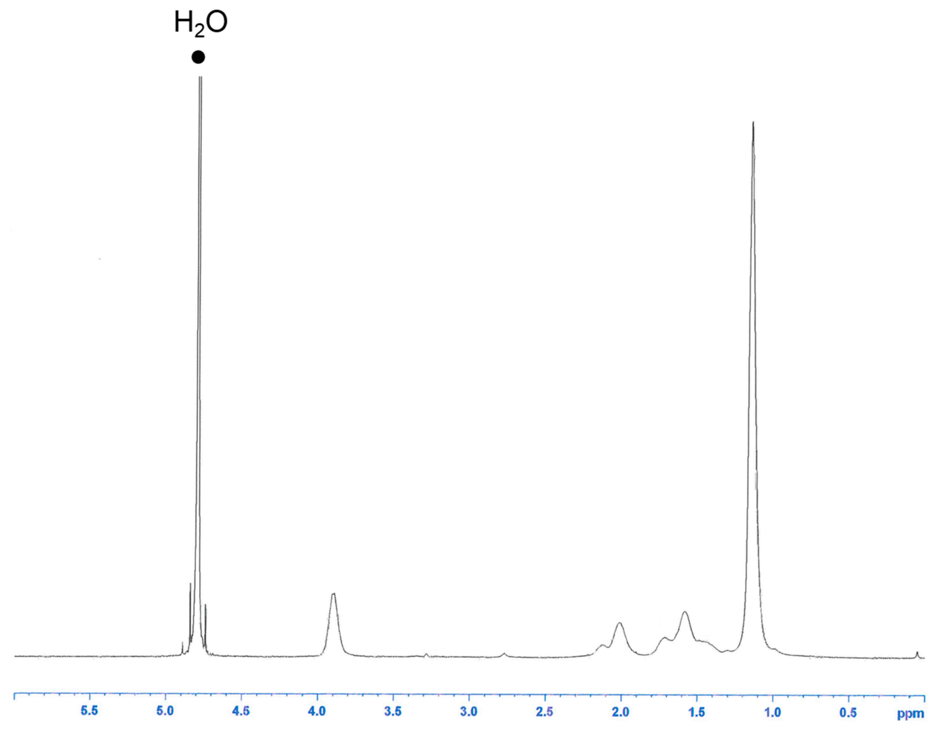

NANOGEL-**6**

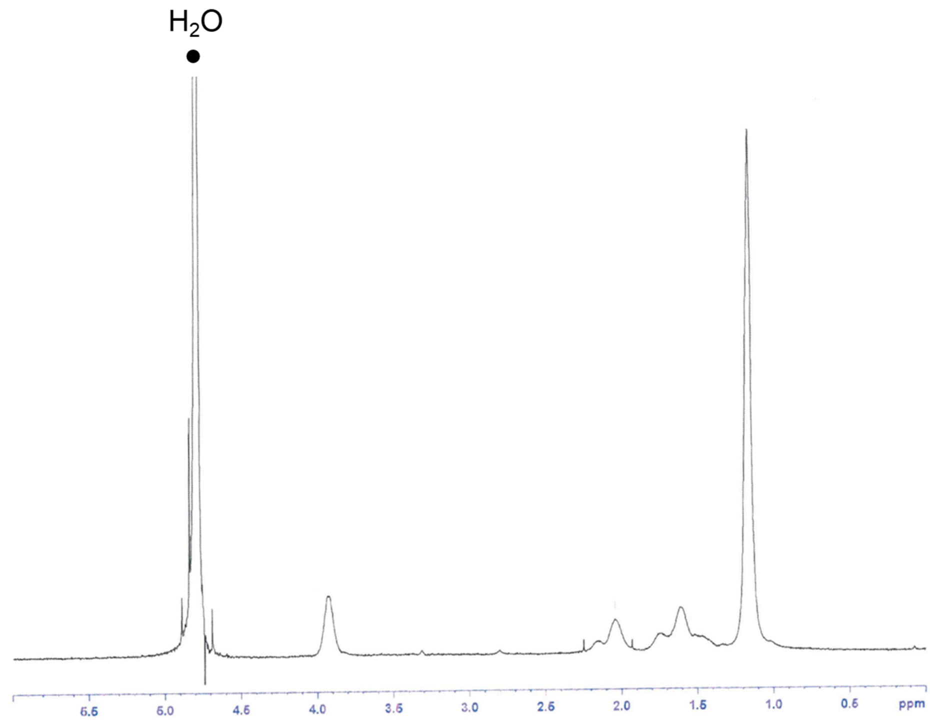

Figure S1. (continued)

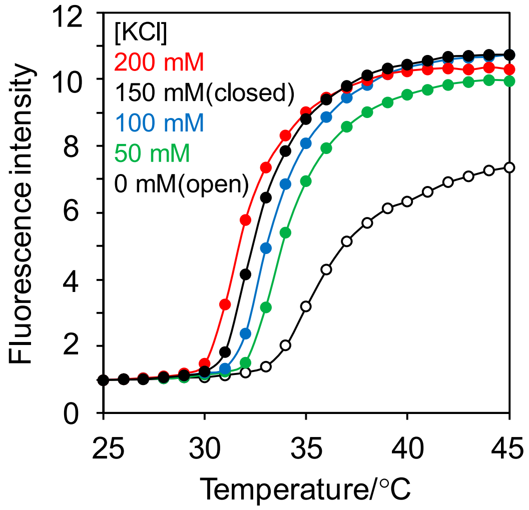

Figure S2. Effects of the KCl concentration on fluorescence responses of NANOGEL-**3** as a representative. The fluorescence intensity is normalized at 25 °C. The samples were excited at 456 nm.
